# Behavioral strategies during incubation influence nest and female survival of Wild Turkeys

**DOI:** 10.1002/ece3.6812

**Published:** 2020-09-24

**Authors:** Ashley K. Lohr, James A. Martin, Gregory T. Wann, Bradley S. Cohen, Bret A. Collier, Michael J. Chamberlain

**Affiliations:** ^1^ Warnell School of Forestry and Natural Resources University of Georgia Athens GA USA; ^2^ College of Arts and Sciences Tennessee Technological University Cookeville TN USA; ^3^ School of Renewable Natural Resources Louisiana State University Agricultural Center Baton Rouge LA USA

**Keywords:** incubation, *Meleagris gallopavo*, nest survival, recess, reproduction, wild turkey

## Abstract

Females must balance physiological and behavioral demands of producing offspring with associated expenditures, such as resource acquisition and predator avoidance. Nest success is an important parameter underlying avian population dynamics. Galliforms are particularly susceptible to low nest success due to exposure of ground nests to multiple predator guilds, lengthy incubation periods, and substantive reliance on crypsis for survival. Hence, it is plausible that nesting individuals prioritize productivity and survival differently, resulting in a gradient of reproductive strategies. Fine‐scale movement patterns during incubation are not well documented in ground‐nesting birds, and the influence of reproductive movements on survival is largely unknown. Using GPS data collected from female wild turkeys (*n* = 278) across the southeastern United States, we evaluated the influence of incubation recess behaviors on trade‐offs between nest and female survival. We quantified daily recess behaviors including recess duration, recess frequency, total distance traveled, and incubation range size for each nest attempt as well as covariates for nest concealment, nest attempt, and nest age. Of 374 nests, 91 (24%) hatched and 39 (14%) females were depredated during incubation. Average nest survival during the incubation period was 0.19, whereas average female survival was 0.78. On average, females took 1.6 daily unique recesses (*SD* = 1.2), spent 2.1 hr off the nest each day (*SD* = 1.8), and traveled 357.6 m during recesses (*SD* = 396.6). Average nest concealment was 92.5 cm (*SD* = 47). We found that females who took longer recess bouts had higher individual survival, but had increased nest loss. Females who recessed more frequently had lower individual survival. Our findings suggest behavioral decisions made during incubation represent life‐history trade‐offs between predation risk and reproductive success on an unpredictable landscape.

## Graphical abstract

We found that female wild turkeys who took longer recess bouts had higher individual survival, but had increased nest loss. Females who recessed more frequently had lower individual survival. Our findings suggest behavioral decisions made during incubation represent life‐history trade‐offs between predation risk and reproductive success.

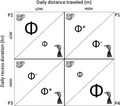

## 1 INTRODUCTION

Reproduction is an energetically costly behavior necessary for population viability and genetic exchange (Avise, 1996). Female investment in producing offspring versus. individual growth and maintenance is governed by resource allocation theory, which states resources put toward one life‐history trait (e.g., survival) cannot simultaneously be put toward another (e.g., reproduction; Audzijonyte & Richards, 2018; Boggs, 1992). Hence, females balance energetic demands of producing offspring against resource acquisition and predator avoidance (Boggs, 1992; Kie, 1999). As species evolve under various degrees of predation pressure (Lamanna & Martin, 2016; Martin, 1995), predator‐rich environments have driven evolution of diverse life‐history strategies, such as bet hedging to reduce temporal variance in individual fitness (Einum & Fleming, 2004; Fontaine et al., 2007; Fontaine & Martin, 2006; Simovich & Hathaway, 1997). In unpredictable environments, bet hedging may involve prioritizing individual survival over producing offspring to ensure future reproductive opportunities (Cohen, 1966, 1967; Danforth, 1999; Simovich & Hathaway, 1997). Within avian taxa, mortality of females during nesting shapes reproductive strategies (Fontaine & Martin, 2006; Ricklefs, 1969), and failure to respond to predation risk produces negative fitness consequences. Thus, individuals likely prioritize productivity and survival differently, resulting in a gradient of reproductive strategies (Afton, 1980; Jones, 1989).

Nest success is an important parameter underlying avian population dynamics (Ricklefs, 1969; Sæther & Bakke, 2000). Gallinaceous birds are particularly susceptible to nest loss due to exposure of ground nests to multiple predator guilds, lengthy incubation periods, and substantive reliance on crypsis for survival (Blomberg et al., 2015). Furthermore, female‐only incubation is common in galliform species, making females especially vulnerable to predation (Cockburn, 2006; Johnsgard, 1983). For these reasons, wild turkeys (*Meleagris gallopavo*) are an ideal gallinaceous bird in which to examine reproductive behaviors and life‐history trade‐offs. Female turkeys have particularly lengthy incubation periods, ranging from 25 to 30 days (Conley et al., 2015; Healy, 1992). In the southeastern United States, numerous predator species depredate nests (Dreibelbis et al., 2008; Lehman et al., 2008; Martin et al., 2015; Miller & Leopold, 1992) and adults during incubation periods (Chamberlain & Leopold, 1999; Hubbard et al., 1999; Moore et al., 2010; Palmer et al., 1993).

Nest placement has long been thought to be the primary driver of survival of wild turkey nests. Extensive research has evaluated impacts of vegetation at nest sites on nest success (Wood et al., 2018; Yeldell et al., 2017) and described nest site selection by female turkeys (Fuller et al., 2013; Lehman et al., 2008; Little et al., 2016; Porter, 1992; Streich et al., 2015). However, contemporary works have continued to suggest that vegetation at nest sites may not be the main driver of nest success (Burk et al., 1990; Byrne & Chamberlain, 2013; Lazarus & Porter, 1985; Thogmartin, 1999; Yeldell et al., 2017). Likewise, nest placement may influence female survival because incubating females must balance embryonic development and resource acquisition via recess movements (Williams et al., 1971). Extensive literature on a suite of avian species suggests nest attentiveness is influenced by predation risk, egg cooling, and female body condition (Haftorn, 1988; MacDonald et al., 2013; Weathers & Sullivan, 1989; Wiebe & Martin, 1997, 2000). For wild turkeys, recess bouts are thought to reduce disturbance near the nest and allow incubating females to defecate and forage away from the nest. However, recess behaviors in turkeys are poorly understood and based on sporadic observations of birds during the incubation period (Conley et al., 2015; Green, 1982; Williams et al., 1971). Notably, contemporary works using fine‐scale movements to detail recess behaviors have either been hampered by modest samples sizes (Conley et al., 2015), or detailed average recess behaviors for each nest attempt (Bakner et al., 2019), rather than seeking to identify consequences of daily recess behaviors on individual fitness metrics. Moreover, previous studies did not consider consequences of recess behaviors to the female.

To expand upon earlier (Green, 1982; Williams et al., 1971) and more contemporary works describing incubation recess behaviors of wild turkeys (Bakner et al., 2019; Conley et al., 2015), our objectives were to (a) examine daily recess behaviors of incubating female Eastern wild turkeys and (b) relate incubation behaviors and nest concealment to nest and female survival to identify whether individual females used strategies to maximize nest success or survival. We hypothesized incubating females would prioritize productivity and survival individually, which would be reflected in differences in daily movements and space use.

**FIGURE 1:**
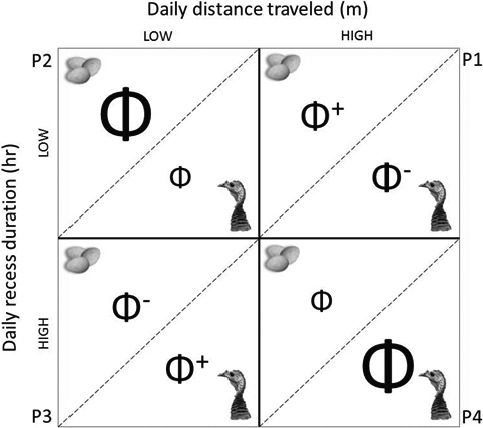
Confusion matrix illustrating predicted effects of daily distance traveled (m) and daily recess duration (hr) on survival of nests and individual female wild turkeys (*Meleagris gallopavo*). Large Φ represents a high survival probability under the specified parameter intensities, small Φ denotes a low survival probability, and Φ^+^ and Φ^−^ correspond to intermediate survival probabilities

We generated a confusion matrix illustrating the predicted effects of daily distance traveled and daily recess duration (including frequency of recesses) on nest and female survival because these covariates best reflected nest attentiveness and movements proximal to a nest (Figure 1). We assumed that vegetation was indirectly linked to potential effects of daily movements on survival (Φ) and therefore did not include nest concealment in the matrix. Large Φ represented a high survival probability under the specified parameters, small Φ denoted a low survival probability, and Φ^+^ and Φ^−^ corresponded to intermediate survival probabilities. Under average conditions, we predicted a female would either reduce daily movements while spending more time off the nest at the expense of the nest (P3), or spend more time incubating but increase daily movements at the expense of the female (P1). Both scenarios allowed a female to balance resource acquisition, embryonic development, and predator avoidance (Boggs, 1992; Jones, 1989; Kie, 1999). Considering turkeys are a long‐lived species capable of renesting multiple times each reproductive season (Wood et al., 2018; Yeldell et al., 2017), we predicted a female would spend more time off the nest and increase daily movements (P4) if she perceived direct (i.e., predator) or indirect (i.e., environmental) threats to her survival (Ghalambor & Martin, 2001). This may demonstrate a bet‐hedging strategy used by species with long incubation periods and high adult survival outside the nesting season (Ghalambor & Martin, 2001; Martin, 2002). Bet‐hedging behaviors have obvious negative repercussions for nest success, but increase the likelihood a female will survive to renest later that season or in a successive breeding season (Matysioková & Remeš, 2018; Wiebe & Martin, 2000). Lastly, if a female prioritized nest survival over self‐maintenance or perceived low predation risk, she would spend more time incubating and reduce her daily movements (P2; Fontaine & Martin, 2006).

## 2 METHODS

### 2.1 Study areas

**FIGURE 2:**
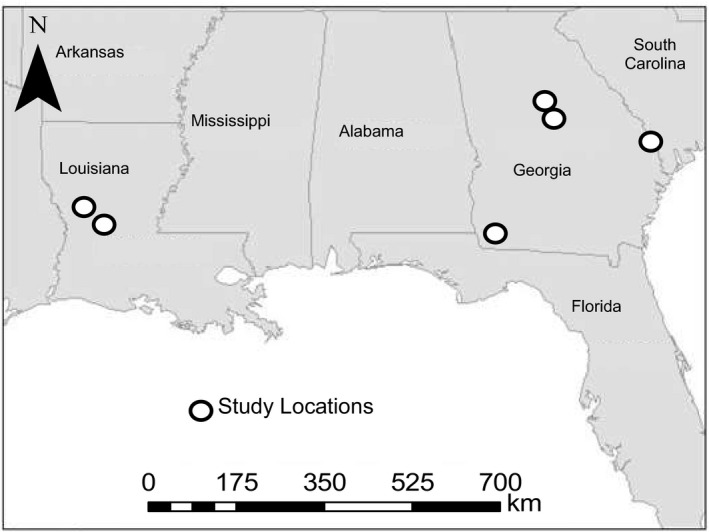
Map of study sites in the southeastern United States where incubation recess behaviors were evaluated for female wild turkeys (*Meleagris gallopavo*) during 2014–2018. The symbol in South Carolina represents 3 study sites, collectively known as the Webb WMA Complex

We conducted research on 8 study sites and surrounding privately owned land in 3 states located in the southeastern United States (Figure 2). The study sites consisted predominantly of mixed pine‐hardwood forests managed with dormant and growing‐season prescribed fire. Specifically, we conducted research on 2 sites in west‐central Louisiana, Kisatchie National Forest (KNF) and Peason Ridge Wildlife Management Area (WMA). The KNF was owned and managed by the United States Forest Service (USFS), whereas Peason Ridge WMA was owned and managed by the United States Army. These sites consisted of pine‐dominated forests, hardwood riparian zones, and forested wetlands, with forest openings, utility right‐of‐ways, and forest roads distributed throughout. Dominant overstory species included longleaf pine (*Pinus palustris*), loblolly pine (*P. taeda*), oaks (*Quercus* spp.), hickories (*Carya* spp.), red maple (*Acer rubrum*), and sweetgum (*Liquidambar styraciflua*). Prescribed fire was applied on a 3‐ to 5‐year return interval. For a detailed description of site conditions on KNF and Peason Ridge WMA, see Yeldell et al. (2017).

We also conducted research on 3 sites in Georgia: Cedar Creek, B. F. Grant, and Silver Lake WMAs. Silver Lake WMA, located in southwest Georgia, was owned and managed by the Georgia Department of Natural Resources—Wildlife Resources Division (GADNR). Silver Lake WMA was comprised of mature pine forests and forested wetlands. Overstory species were predominantly longleaf pine, loblolly pine, slash pine (*P. elliottii*), oaks, and sweetgum. Prescribed fire was applied on a 2‐ to 3‐year return interval. For a detailed description of site conditions on Silver Lake WMA, see Wood et al. (2018).

Cedar Creek and B. F. Grant WMAs were both located in the Piedmont region of Georgia. Cedar Creek WMA was owned by the U. S. Forest Service and managed in partnership with GADNR. Cedar Creek WMA was composed primarily of upland loblolly pine stands, mixed pine‐hardwood forests, and hardwood lowlands dominated by oaks, sweetgum, and hickories. B. F. Grant WMA was owned by the Daniel B. Warnell School of Forestry and Natural Resources at the University of Georgia and was managed cooperatively by the GADNR and the Warnell School. B. F. Grant WMA consisted primarily of loblolly pine stands, agricultural fields, mixed pine‐hardwood forests, and hardwood bottoms similar in composition to Cedar Creek. Agricultural fields were mainly grazed mixed fescue (*Festuca* spp.) and hay fields planted for rye grass (*Lolium* spp.). Utility right‐of‐ways and forest roads were found throughout both study sites, and prescribed fire was applied on both sites on a 3‐ to 5‐year return interval. Much of the private land surrounding these WMAs was subject to intensive timber harvest regimes.

Lastly, we conducted research on 3 contiguous WMAs (Webb, Hamilton Ridge, and Palachucola, hereafter Webb WMA Complex) in southeastern South Carolina, all managed by the South Carolina Department of Natural Resources (SCDNR). The Webb WMA Complex consisted of longleaf, loblolly, and slash pine forests as well as hardwood stands along riparian corridors and bottomland hardwood wetlands. Prescribed fire was applied on a 3‐ to 5‐year return interval. For a detailed description of site conditions on the Webb WMA Complex, see Wightman et al. (2018).

### 2.2 Turkey capture and processing

We captured female turkeys using rocket nets from January to March 2014–2018. Captured individuals were aged using the presence (adult) or absence (juvenile) of barring on the ninth and tenth primary feathers (Pelham & Dickson, 1992). We banded each bird with a serially numbered butt‐end style or riveted aluminum tarsal band (National Band and Tag Company, Newport, Kentucky, USA) and radio‐tagged each female with a backpack‐style, mortality‐sensitive GPS transmitter with VHF capabilities (Biotrack Ltd., Wareham, Dorset, UK; Guthrie et al., 2011). We programmed transmitters to record hourly locations from 05:00–20:00 and one nightly location at 23:59 for the life of the unit or until the unit was recovered (Cohen et al., 2018). All birds were released at the capture location immediately following processing. Turkey capture, handling, and marking procedures were approved by the Institutional Animal Care and Use Committee at the University of Georgia (Protocol #A2014 06008Y1A0, A343701, and A2016 04‐001‐R1) and the Louisiana State University Agricultural Center (Protocol #A2014‐013 and A2015‐07).

### 2.3 Nest monitoring

We used a handheld, 3‐element Yagi antenna and receiver (Advanced Telemetry Systems Inc., Isanti, Minnesota, USA) to monitor survival and reproductive activity of all radio‐tagged females. We downloaded GPS locations from each female ≥1 time per week and assumed onset of incubation when GPS locations were fixed around a central point for at least 24 hr (Yeldell et al., 2017). We monitored incubating females daily using radio telemetry, and once incubation was terminated, we located the nest using GPS coordinates to determine nest fate and recorded nest site characteristics. We considered nests successful if ≥1 egg hatched (Conley et al., 2016). We continued to monitor females for additional nest attempts until reproductive activity ceased.

### 2.4 Vegetation sampling at nest sites

Because nest concealment may influence nest and female survival (Fuller et al., 2013; Lehman et al., 2008; Nguyen et al., 2004), we conducted vegetation surveys at each nest site at expected date of hatch regardless of nest fate (Gibson et al., 2016; McConnell et al., 2017). We estimated lateral visual obstruction (cm) by placing a 2‐m tall Robel pole (Robel et al., 1970) at the nest bowl and recording minimum vegetation height readings from 15 m away in each cardinal direction, as this encompassed the vegetation conditions immediately surrounding the nest that we deemed relevant to wild turkeys and predators potentially encountering nests (Wood et al., 2018; Yeldell et al., 2017). We then averaged the visual obstruction readings to generate one value at each nest site.

### 2.5 Incubation analysis

To isolate incubation behaviors from pre‐ and postnesting movements, we censored the first and last days of incubation (Conley et al., 2015). To account for potential GPS error (Guthrie et al., 2011) and short movements away from the nest that did not constitute recess movements, we placed a 27.5 m buffer around each nest as detailed in Collier et al. (2019). Following Collier et al. (2019) and Bakner et al. (2019), we defined recess movements as any GPS location >27.5 m from the nest coordinates, whereas GPS locations ≤27.5 m from the nest coordinates were considered as nest (incubation) locations. Previous studies have noted that space use may influence survival and reproductive success (Badyaev et al., 1996; Patrick & Weimerskirch, 2017; Yoder et al., 2004), and movements to and from nests may increase predation risk to parents and offspring (Martin, 2002; Wiebe & Martin, 1997). Hence, we sought to quantify incubation recess behaviors such as daily range size and daily movements. We defined a unique recess as ≥1 GPS location >27.5 m from the nest coordinates prior to a female returning to the nest. We determined recess duration as the total number of GPS locations that fell outside of the nest buffer each day. Using R v3.4.1 (R Core Team, 2019), we measured total daily distance traveled, number of daily unique recesses, daily recess duration, and daily range size for each nest attempt. We used dynamic Brownian Bridge Movement Models (hereafter, dBBMM) to quantify 99% daily utilization distributions, using a window size of 7, margin of 3, and location error of 20 m (Cohen et al., 2018; Kranstauber et al., 2012). We performed all utilization distribution calculations using R package move (Kranstauber et al., 2017).

### 2.6 Nest survival model

We constructed a Bayesian hierarchical nest survival model (Royle & Dorazio, 2008) using the R2jags package (Su & Yajima, 2015) in R (R Core Team, 2019) to estimate nest survival. We parameterized models using covariates likely to influence survival of nests (Bakner et al., 2019; Lehman et al., 2008; Wiebe & Martin, 1997), which included daily distance traveled, unique recesses taken daily, recess duration, daily range size, and nest concealment. For most precocial avian species, older nests are more likely to survive because nests in riskier locations are depredated early (Klett & Johnson, 1982). Hence, we also included nest attempt and nest age to assess their effects on nest survival (Wilson et al., 2007). We estimated period survival as daily survival expanded for the entire 30‐day incubation cycle (Shaffer & Thompson, 2007). To avoid introducing bias into our period survival estimates, we included censored nests and females in our period survival estimates. However, for the analysis of covariate effects we decided to censor nests incubated <3 days since we were unable to isolate incubation behaviors from nests of such short duration. Also, for nests included in the covariate analyses, we used the mean of each covariate in the model for the first and last day of the exposure period because movement metrics were poorly estimated those days. To examine collinearity, we calculated Pearson correlations (*r*) for all pairs of predictor variables. We ultimately removed daily range size from our models due to a positive correlation with daily distance traveled (*r* = 0.74; Dormann et al., 2013). We used methodology outlined by Kruschke (2018) and Makowski et al. (2019) to develop decision rules using Bayesian posterior probabilities as a basis to determine the statistical significance of covariates on period survival rates. We developed 95% highest density intervals (credible intervals) that provided indices of uncertainty. We then computed the probability of direction (*pd*) which provided the probability that each covariate either positively or negatively influenced nest and female survival. We compared posterior estimates of effects of each covariate on nest and female survival using *pd* values. We interpreted values close to 0.5 to suggest no biological effect of covariates, but values ≥0.9 as biologically significant (Loman et al., 2018; Ruiz‐Gutiérrez et al., 2010).

As per Royle and Dorazio (2008), we treated nest fate between successive days as the sampling unit. We designated nest attempt *i* on a given day of incubation *j* as 1 for an active nest and 0 for a nest that had been depredated or otherwise failed. We treated the probability of nest survival from day *j* to day *j + 1* as a Bernoulli distribution. Because our study included wild Turkeys monitored across multiple study sites and years, we specified site and year as random effects with site nested within year. We chose uninformative priors by specifying distributions for both model coefficients and site and year random effects as Normal(0, 0.001), where 0 and 0.001 are the distribution's mean and precision (1/*σ* ^2^), respectively. We then fit the following model using nest survival covariates on the logit scale:

$$\begin{array}{cc} \text{logit}\left(\Phi_{i,j}\right) & =\beta_{0}+\beta_{1}*{\text{attempt}}_{i}+\beta_{2}*{\text{conceal}}_{i}+\beta_{3}*{\text{distance}}_{i,j}+\beta_{4}*{\text{duration}}_{i,j}\\ & +\beta_{5}*{\text{recess}}_{i,j}+\beta_{6}*{\text{nage}}_{i,j}+{\text{Year}}_{i}+{\text{Site}}_{i} \end{array}$$

where attempt*_i_* and nage*_i,j_* represented the effects of nest attempt and nest age on nest survival, respectively. Conceal*_i_* characterized the effect of nest concealment on survival, distance*_i,j_* symbolized the effect of daily distance traveled on nest survival, duration*_i,j_* denoted the effect of daily recess duration on nest survival, and recess*_i,j_* represented the effect of daily recess frequency on nest survival. Temporal and spatial random effects were denoted by Year*_i_* and Site*_i_*.

### 2.7 Female survival model

We used the R2jags package in R (R Core Team, 2019) to generate an additional Bayesian hierarchical model to estimate female survival. With the exception of nest age, we used the same parameters as the nest survival model because those covariates likely influence female survival during incubation (Dudko et al., 2019; Lehman et al., 2008; Martin, 2002). Following Royle and Dorazio (2008), we treated female fate between successive days as the sampling unit. We designated individual female *i* on a given day of incubation *j* as 1 for alive and 0 for a female that had been depredated. The probability of female survival from day *j* to day *j + 1* was modeled using a Bernoulli distribution. We then built the following model using female survival covariates on the logit scale:

$$\begin{array}{cc} \text{logit}\left(\Phi_{i,j}\right) & =\beta_{0}+\beta_{1}*{\text{attempt}}_{i,j}+\beta_{2}*{\text{conceal}}_{i,j}+\beta_{3}*{\text{distance}}_{i,j}+\beta_{4}*{\text{duration}}_{i,j}\\ & +\beta_{5}*{\text{recess}}_{i,j}+{\text{Year}}_{i}+{\text{Site}}_{i} \end{array}$$

For both the nest and female survival models, we used Markov chain Monte Carlo (MCMC) to estimate posterior distributions of the model parameters. We conducted simulations using 3 chains, 4,000 iterations, a burn‐in value of 1,000, and a thinning rate of 3 for the nest survival model, whereas we used 7,000 iterations and a burn‐in value of 2,000 for the female survival model (Gelman & Rubin, 1992). All estimated parameters had R‐hat values <1.1, meaning all chains converged (Gelman et al., 2004).

## 3 RESULTS

**FIGURE 3:**
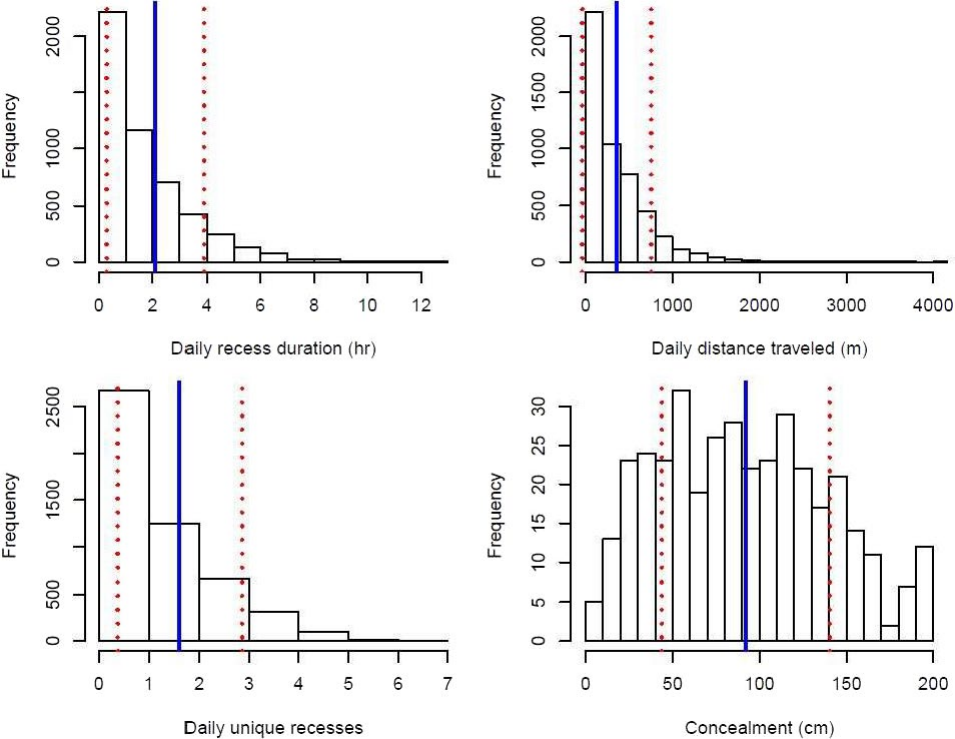
Histograms illustrating the range of observed values for 4 covariates used to model nest and female wild turkey (*Meleagris gallopavo*) survival. Solid blue lines represent $\bar{x}$ and dashed red lines indicate ± 1 standard deviation. Females spent 0–9 hr off the nest each day ($\bar{x}$ = 2.09, *SD* = 1.80), took 0–7 daily unique recesses ($\bar{x}$ = 1.62, *SD* = 1.24), and traveled 0–4,103 m in a day ($\bar{x}$ = 357.63 m, *SD* = 396.58). Values of nest concealment ranged from 8.75–200 cm ($\bar{x}$ = 92.5 cm, *SD* = 47)

We used 374 nests (262 initial attempts, 90 s attempts, 20 third attempts, 2 fourth attempts) incubated by 278 female wild Turkeys (248 adults, 30 juveniles) during 2014–2018 for nest and female survival analyses. Prior to covariate analysis, we removed 32 nests that were incubated <3 days since we were unable to isolate incubation behaviors from nests of such short duration. We observed earliest onset of incubation on 18 March and last date of termination on 20 July, resulting in an incubation season spanning 124 days. Of 374 nests, 91 (24%) hatched and 39 (14%) females were depredated during incubation. Based on GPS data and anecdotal evidence detailing the presence of feathers or a carcass proximal to the nest bowl, we inferred that 38 of 39 females were killed at the nest site. Average daily and period nest survival rates were 0.95 (95% CrI = 0.91, 0.97) and 0.20 (95% CrI = 0.07, 0.37), respectively, whereas average daily and period female survival rates were 0.99 (95% CrI = 0.985, 0.995) and 0.78 (95% CrI = 0.639, 0.872), respectively. On average, females took 1.62 daily unique recesses (*SD* = 1.24), spent 2.09 hr off the nest each day (*SD* = 1.80), and traveled 357.63 m in a day (*SD* = 396.58; Figure 3). Average nest concealment was 92.5 cm (*SD* = 47; Figure 3).

**FIGURE 4:**
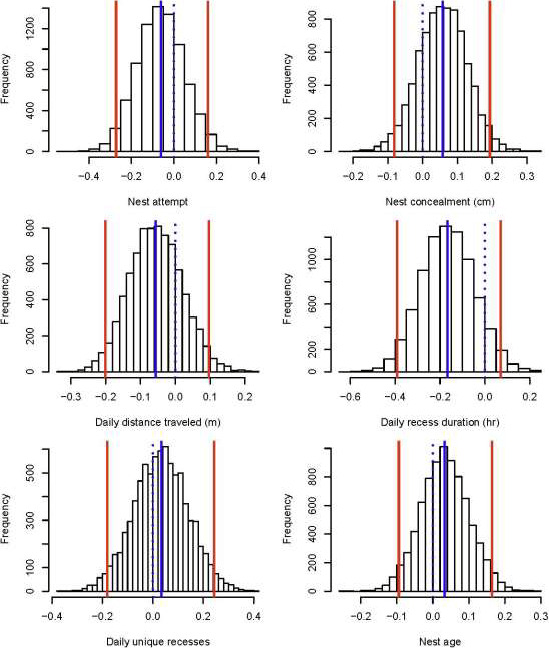
Posterior distributions for covariates used to model daily survival for wild turkey (*Meleagris gallopavo*) nests. Solid blue lines denote µ, red lines represent 95% credible intervals, and dashed blue lines indicate 0

**FIGURE 5:**
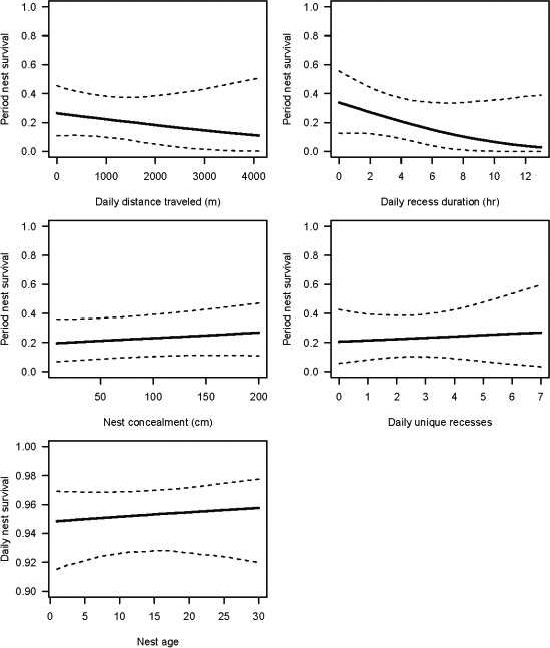
Predicted effects of model covariates on period (30 days) survival probabilities for wild turkey (*Meleagris gallopavo*) nests

The prediction that females would prioritize nest survival over individual survival (P2) was not supported. For nest survival, posterior response to daily recess duration was stronger than all other parameter responses (μ of posterior distribution with 95% credible intervals = −0.17, −0.40 to 0.08; Table 1, Figure 4). We observed that increasing daily recess duration had a 92% probability of negatively influencing nest survival. Specifically, nests were 1.19 times less likely to survive with every 1.8‐hr increase in daily recess duration (Figure 5). There were no effects of nest attempt, nest age, nest concealment, daily distance traveled, or daily unique recesses on nest survival rates (Table 1, Figures 4 and 5).

**TABLE 1:** Posterior means, 95% credible intervals, and probability of direction (*pd*) statistic for covariates used to model daily survival for wild turkey (*Meleagris gallopavo*) nests. Means >0 positively influence daily nest survival, whereas means <0 negatively influence survival

Survival covariates	0.025	0.250	0.500	0.750	0.975	*pd*
Intercept (β_0)	2.51	2.88	3.04	3.21	3.58	
Nest attempt (β_1)	−0.27	−0.14	−0.06	0.01	0.16	0.734
Concealment (β_2)	−0.08	0.01	0.06	0.11	0.19	0.793
Distance traveled (β_3)	−0.20	−0.11	−0.06	0.00	0.10	0.762
Recess duration (β_4)	−0.39	−0.25	−0.17	−0.08	0.07	0.916
Unique recesses (β_5)	−0.18	−0.04	0.03	0.11	0.24	0.617
Nest age (β_6)	−0.09	−0.01	0.03	0.08	0.17	0.699

**FIGURE 6:**
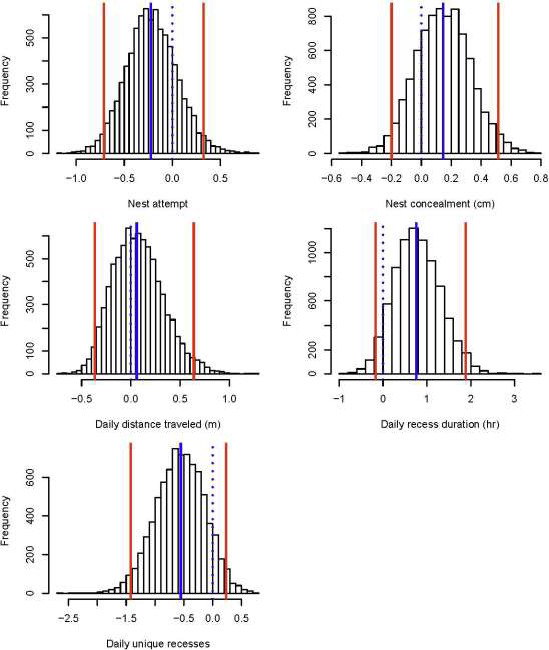
Posterior distributions for covariates used to model daily survival for female wild turkeys (*Meleagris gallopavo*). Solid blue lines denote µ, red lines represent 95% credible intervals, and dashed blue lines indicate 0

**FIGURE 7:**
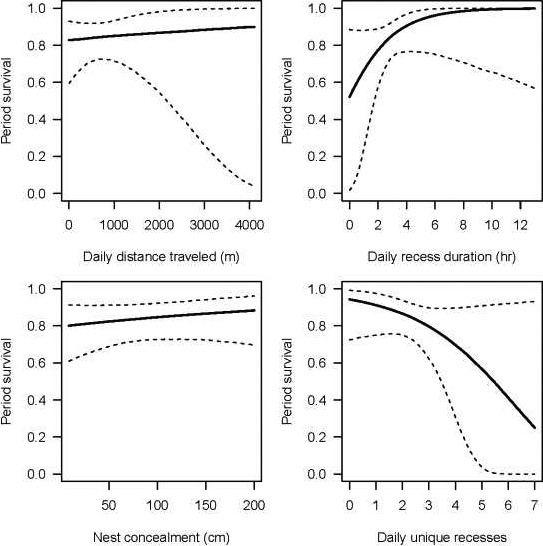
Predicted effects of model covariates on period (30 days) survival probabilities for female wild turkeys (*Meleagris gallopavo*)

In general, the prediction that females would prioritize individual survival over nest survival (P4) was supported. For female survival, posterior responses were strongest for daily recess duration (μ of posterior distribution with 95% credible intervals = 0.79, −0.17 to 1.88; Table 2, Figure 6) and number of daily recesses (μ of posterior distribution with 95% credible intervals = −0.56, −1.42 to 0.23; Table 2, Figure 6). We observed that increasing daily recess duration had a 95% probability of positively influencing female survival, whereas increasing numbers of daily recesses had a 93% probability of negatively influencing female survival. Specifically, incubating females were 2.14 times more likely to survive with every 1.8‐hr increase in daily recess duration (Figure 7). Conversely, odds of survival for incubating females were 1.74 times less likely as number of daily recesses increased by 1.24 (Figure 7). There were no significant effects of nest attempt, nest concealment, or daily distance traveled on female survival (Table 2, Figures 6 and 7).

**TABLE 2:** Posterior means, 95% credible intervals, and probability of direction (*pd*) statistic for covariates used to model daily survival for female wild turkeys (*Meleagris gallopavo*). Means > 0 positively influence daily female survival, whereas means < 0 negatively influence survival

Survival covariates	0.025	0.250	0.500	0.750	0.975	pd
Intercept (β_0)	4.38	5.06	5.39	5.70	6.37	
Nest attempt (β_1)	−0.71	−0.39	−0.22	−0.04	0.33	0.788
Concealment (β_2)	−0.20	0.02	0.15	0.27	0.51	0.788
Distance traveled (β_3)	−0.37	−0.11	0.06	0.24	0.64	0.596
Recess duration (β_4)	−0.17	0.41	0.76	1.14	1.88	0.953
Unique recesses (β_5)	−1.42	−0.85	−0.55	−0.27	0.23	0.928

## 4 DISCUSSION

Recess movements enable incubating birds to balance embryonic development with resource acquisition and predator avoidance (Wiebe & Martin, 2000; Williams et al., 1971). Hence, recess behaviors may bear important implications to fitness. Extant literature on wild turkey incubation behaviors is based on observations of females leaving or returning to nests (Green, 1982; Williams et al., 1971), and only recently have we gained the ability to thoroughly describe recess behaviors (Conley et al., 2015). Recently, Bakner et al. (2019) used a subsample of females monitored during our study to more coarsely assess influences of incubation behaviors on nest survival. The authors noted that cumulative distances traveled per day during incubation most influenced nest survival. We refined the approaches taken by Bakner et al. (2019) by relating daily incubation behaviors to nest and female survival, while also using a larger sample of females monitored across a broader temporal period. Our findings support the prediction that female wild turkeys use multiple strategies during incubation, presumably driven by life‐history trade‐offs between predation risk and reproductive success on a dynamic landscape. Collectively, we found that recess duration and recess frequency had the strongest effects on nest and female survival. Poor period nest survival combined with high female mortality at the nest site suggests that females may be altering their incubation behaviors to prioritize individual survival and ensure future reproductive opportunities.

We observed daily recess duration influenced daily survival of both nests and females. Shorter daily recess bouts correspond to increased nest attentiveness which may make incubating females more susceptible to predation, particularly by predators that rely on olfactory cues to locate prey (Hubbard et al., 1999; Isaksson et al., 2007; Martin et al., 2015). Alternatively, longer daily recess bouts result in unattended nests and may increase nest predation risk. For example, Smith et al. (2012) observed a positive relationship between nest predation and proportion of time shorebirds left nests unattended. Prolonged recess bouts may also slow embryonic development, increase incubation periods, and lengthen nest exposure times (Haftorn, 1988; Lyon & Montgomerie, 1985; MacDonald et al., 2013). Incubation strategies featuring longer daily recess durations may indicate females perceive heightened individual predation risk and therefore prioritize individual survival to ensure future reproductive opportunities, either by renesting later in the season or postponing reproduction until the subsequent nesting season (Fontaine & Martin, 2006; Lima, 2009; Milonoff, 1989; Philippi & Seger, 1989).

Considering most females were killed at their nests, movements away from a nest may enable incubating females to obtain resources while avoiding predation (Eggers et al., 2005). This behavior supports the positive relationship we observed between daily distance traveled and female survival. However, such movements negatively influenced nest survival, most likely due to decreased nest attendance (Lecomte et al., 2009; Smith et al., 2012), although the predicted effects of daily distance moved were not as biologically relevant compared to daily recess duration. Increased daily movements could reflect females traveling to distant foraging sites or prolonged movements proximal to a nest. Movements close to a nest may be indicative of poor female body condition or nest guarding tactics in the event of a threat, such as a snake or mammalian mesopredator that a female turkey could effectively deter (Dreibelbis et al., 2008; Hakkarainen et al., 2002; Martindale, 1982; Marzluff, 1985). Conversely, distant recesses may reflect a lack of resources near the nest (Criscuolo et al., 2000; Lecomte et al., 2009) or heightened perceived predation risk, although the latter is not understood.

Instances of egg depredation and nest loss may be associated with female movements to and from nests (Spaans et al., 2007; Wiebe & Martin, 1997). We noted that number of daily unique recesses had no effect on nest survival, but had a noticeable negative effect on female survival. Frequent, direct movements to and from nest sites likely attract predators observing parental activity or increase numbers of scent trails that guide predators to a nest (Erikstad, 1986; Storaas & Wegge, 1997; Weathers & Sullivan, 1989). Hence, females taking numerous recesses per day may dampen population productivity over time due to decreases in female survival (see Collier et al., 2009).

Previous authors have found that nests attempted later in the season may have a higher probability of hatching due to a lower density of nests on the landscape, improved vegetation cover when compared to the onset of the nesting season, and increased availability of alternative food sources (Lehman et al., 2008; Lockwood & Sutcliffe, 1985; Norman et al., 2001; Rumble & Hodorff, 1993). Munkebye et al. (2003) observed greatest rates of nest predation in willow ptarmigan (*Lagopus lagopus*) immediately before the first nest hatched, and nest predation rate increased with increasing numbers of available clutches. However, we found that nest age and nest attempt were not important predictors of nest or female survival. Bakner et al. (2019) noted that most turkey nests failed within 14 days of incubation, and we observed low nest survival regardless of nest age. Increased nest failure regardless of nest initiation date may be due to increased predator densities (Coates & Delehanty, 2010; Johnson et al., 1989; Keith, 1961), and the overall low nest success we observed suggests that turkeys are nesting on a predator‐rich landscape.

Trade‐offs between nest and female survival may exist as nest concealment increases. Wiebe and Martin (1998) observed that white‐tailed ptarmigan (*Lagopus leucura*) nests placed in areas with increased cover were less likely to be depredated, but incubating adults were more vulnerable to mammalian predators. We observed that nest concealment, described as a measure of vegetation obstruction on the projected hatch date of each nest attempt, had no apparent effect on nest or female survival. Previous research has detailed the significance of understory conditions, such as vegetation height and stem density, to nest placement and nest success (Conley et al., 2015; Fuller et al., 2013; Lehman et al., 2008; Little et al., 2016; Streich et al., 2015), although there are notable inconsistencies among studies in regards to which vegetation characteristics, if any, most influence nest success or survival (Byrne & Chamberlain, 2013; Yeldell et al., 2017). We offer that vegetation obstruction, as we measure it, may not be an important metric influencing nest or female survival (Burk et al., 1990; Lazarus & Porter, 1985; Storaas & Wegge, 1997; Thogmartin, 1999).

Our findings suggest that female wild turkeys exhibit incubation strategies that represent trade‐offs between predation risk and reproductive success. Landscapes featuring an abundance of nest predators may favor longer incubation bouts (via reduced daily recess duration) and few daily unique recesses to reduce activity around the nest and increase nest attendance (Coates & Delehanty, 2008; Smith et al., 2012; Wiebe & Martin, 1997). Alternatively, if larger predators capable of taking females are abundant, incubating females likely benefit from taking few but significantly longer daily unique recesses (Conway & Martin, 2000). In North America, predator richness increases at southern latitudes (Sandom et al., 2013; Wilson, 1974), and predator guilds within the southeastern United States have changed over the course of the last half‐century (Hill et al., 1987; Lovell et al., 1998). Given this increase in predator diversity, it may be challenging for turkeys to balance such opposing incubation strategies. Additionally, turkeys live in a stochastic environment and many predation events, particularly nest depredations, may be a result of opportunistic foraging (Byrne & Chamberlain, 2015; Storaas & Wegge, 1997). Therefore, it is plausible that turkeys have not yet developed an adequate ability to perceive this elevated and dynamic predation risk. As predators continue to influence evolution of life‐history traits by placing constraints on recess behaviors (Conway & Martin, 2000; Fontaine et al., 2007), natural selection will begin to favor optimal incubation strategies that ensure future reproductive success. Future studies examining temporal variance in female incubation rhythms, spatial and temporal predator distribution and predation patterns during the nesting season, and plasticity of phenotypic traits (such as clutch and egg size) are necessary to further explore reproductive strategies within wild Turkey populations.

## CONFLICTS OF INTEREST

None.

## AUTHOR CONTRIBUTION

**Ashley Lohr:** Data curation (lead); Formal analysis (supporting); Investigation (supporting); Methodology (supporting); Writing‐original draft (lead). **James Martin:** Formal analysis (supporting); Methodology (supporting); Writing‐original draft (supporting). **Gregory Wann:** Methodology (supporting); Software (supporting); Visualization (supporting). **Bradley Cohen:** Conceptualization (supporting); Funding acquisition (supporting); Writing‐review & editing (supporting). **Bret Collier:** Methodology (supporting); Writing‐review & editing (supporting). **Michael J. Chamberlain:** Conceptualization (supporting); Formal analysis (supporting); Funding acquisition (lead); Investigation (supporting); Methodology (supporting); Project administration (lead); Supervision (lead); Writing‐original draft (supporting); Writing‐review & editing (lead).

## ETHICAL APPROVAL

This research was conducted with approval from the Institutional Animal Care and Use Committee at the University of Georgia (Protocol #A2014 06008Y1A0, A343701, and A2016 04–001‐R1) and the Louisiana State University Agricultural Center (Protocol #A2014‐013 and A2015‐07).

## Data Availability

The data files for nest survival, female survival, and average covariates used in modeling, along with metadata, are available upon request or can be accessed on Dryad (https://doi.10.5061/dryad.d7wm37pzx).
